# PCBs and Diabetes: Pinning Down Mechanisms

**DOI:** 10.1289/ehp.121-a32

**Published:** 2013-01-01

**Authors:** Bob Weinhold

**Affiliations:** Bob Weinhold, MA, has covered environmental health issues for numerous outlets since 1996. He is a member of the Society of Environmental Journalists.

Many factors are suspected in the global surge in type 2 diabetes, among them exposures to toxic chemicals including polychlorinated biphenyls (PCBs). To investigate the potential mechanisms of the type 2 diabetes/PCB connection, a team of investigators studied two coplanar PCBs, PCB-77 and PCB-126 [*EHP* 121(1):105–110; Baker et al.]. They found, via *in vitro* and *in vivo* studies with male mice, that exposure rapidly and significantly disrupted several important biological indicators.

PCBs are long-lasting toxicants that accumulate in fatty tissues and remain prevalent in the bodies of people and wildlife around the world, despite a ban decades ago. Human exposure is primarily dietary or occupational.

In mice fed a low-fat diet, PCB-77 was associated with significant impairment of glucose and insulin tolerance, and PCB-126 significantly impaired insulin tolerance, compared with untreated mice fed the same diet. Further investigation of PCB-77 (which the authors say is more prevalent in food than PCB-126) showed the effects lasted two weeks following cessation of PCB exposure. The investigators also found that plasma of treated animals had significantly increased concentrations of tumor necrosis factor alpha (TNF-α) and interleukin 6 for two weeks after the last dose. These inflammatory cytokines are related to insulin resistance. Notably, mice exposed to PCB-77 exhibited a significant increase in TNF-α in adipose tissue (fat), a well-known site of insulin resistance.

**Figure:**
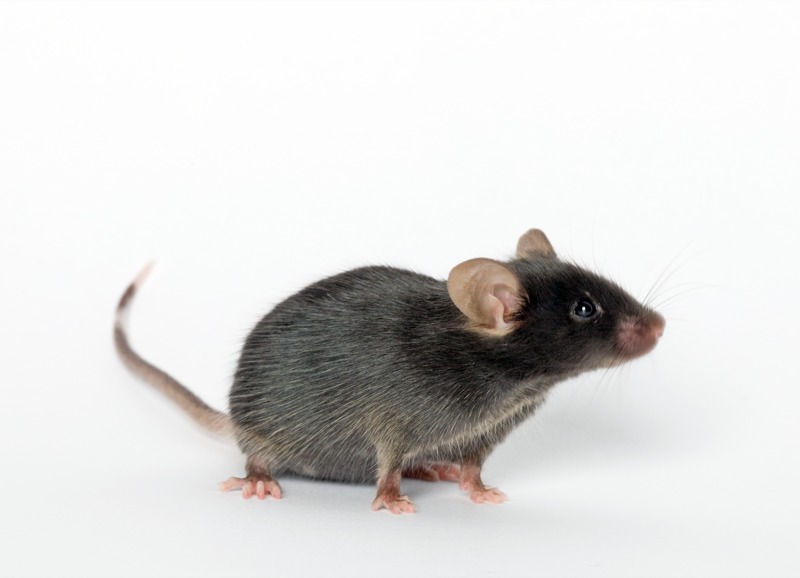
Studies of glucose and insulin tolerance in mice may help clarify the role of PCBs in human diabetes. © The Jackson Laboratory

To explore a process that might be involved in these adverse effects, the team exposed the mice treated with PCB-77 to a known aryl hydrocarbon receptor antagonist and found that the alterations vanished, suggesting this receptor plays a key role in effects on glucose homeostasis. That finding is generally consistent with other studies that have found this pathway plays a role in various bodily responses to PCBs and other toxicants.

In mice fed a high-fat diet, the team found no connection between PCB-77 exposure and effects like those seen in the low-fat group, despite the animals’ having double the concentration of the toxicant in fat tissue. Weight loss improved glucose and insulin tolerance and reduced fat levels of TNF-α in all the mice, but these effects were significantly weaker in exposed mice than in unexposed animals. Previous research has shown that lipid-soluble toxicants sequestered in fat are released into circulation during weight loss, which may reduce PCBs levels in fat but increase them elsewhere in the body.

Before these findings can be translated to humans, larger studies using different exposure lengths and concentrations are needed. Studies should also investigate factors such as the roles of additional PCBs, differences between males and females, and additional possible mechanisms and pathways.

